# Validation of nasal tracheal aspiration in children with lung disease

**DOI:** 10.1186/s12890-022-01992-2

**Published:** 2022-05-17

**Authors:** Louise Østergaard Andersen, Hanne Vebert Olesen, Anne Helene Spannow, Sune Leisgaard Mørck Rubak

**Affiliations:** 1 Department of Paediatrics and Adolescent Medicine, Center of Paediatric Pulmonology and Allergology, Aarhus University Hospital, Skejby, Palle Juul-Jensens Boulevard 99, 8200 Aarhus N, Denmark; 2 Department of Clinical Medicine, Aarhus University, Aarhus N, Denmark

**Keywords:** Paediatrics, Nasal tracheal aspiration, Bronchoalveolar lavage, Cronic lung disease, Cystic fibrosis

## Abstract

**Background:**

Nasal tracheal aspiration (NTA) is a frequently used diagnostic method to assess of infections in the lower airways. However, the validity of the method has not previously been compared to bronchoalveolar lavage (BAL) in non-intubated children with a lung disease. We hypothesised that NTA performed by health professionals using the nares vocal cord distance to be placed at the entrance of the trachea, will result in same finding of bacteria in the lower airways as the gold standard of BAL.

**Methods:**

In a prospective study, 173 paired samples of NTA and BAL were obtained between June 2016 to August 2018. Samples were collected from all patients undergoing bronchoscopy with spontaneous breathing during general anaesthesia. This study compares the microbiological results from the cultures obtained by investigating complete concordance i.e. identical pathogenic bacteria and coherence i.e. absence or presence of pathogenic bacteria growth between NTA and BAL.

**Results:**

Samples were collected in 164 patients, 158 children between 21 days and 18 years of age and six young adults still treated at the paediatric department. The overall similarity (complete agreement) was found in 49% [41–56], sensitivity was 35% [27–45], specificity was 66% [55–76], positive predictive value was 36% [27–46] and negative predictive value was 64% [54–64] concerning complete pathogenic bacteria concordance. If we only considered coherence growth of pathogenic bacteria, similarity was 71% [63–79], sensitivity was 74% [64–81], specificity was 66% [55–76], positive predictive value was 75% [65–82] and negative predictive value was 65% [54–75]. Patients with cystic fibrosis showed a similarity of 88% [73–95], a sensitivity of 92% [76–99], a specificity of 71% [36–95], a positive predictive value of 92% [76–99] and a negative predictive value of 71% [36–95] concerning coherence growth of pathogenic bacteria.

**Conclusion:**

The study indicates that NTA compared to BAL as the gold standard is not clinically useful to assess positive findings of specific bacteria in the lower airway tract. Statistically significantly increased sensitivity and positive predictive value were found in cystic fibrosis patients concerning coherence growth. The clinical usage of NTA remains important as negative findings are of clinical value. However, BAL continues to be preferred as a significantly superior diagnostic tool.

## Background

Investigating children with symptoms of lung and respiratory tract infection is challenging. Finding the precise cause is important to initiate the correct antibiotic treatment to reduce infection and inflammation and thus minimise the bacterial load in the lungs [1]. Previous literature has described several non-invasive “upper airway sampling” methods to obtain information about lower airway tract infection. These methods include oropharyngeal swab, nasopharyngeal swab, nasopharyngeal aspiration, nasotracheal aspiration, oropharyngeal suction, induced sputum, all of which have all been compared to the gold standard bronchoalveolar lavage (BAL) [2–9]. Sampling is performed by inserting the swab into either (1) the nasopharynx through a single nostril or (2) the oropharynx through the mouth until it reaches the posterior pharynx where a gentle rotation is performed for 2–3 s following withdrawal of the swab [2, 3]. Nasopharyngeal aspiration (NPA), nasotracheal aspiration (NTA) or oropharyngeal suction (OPS) is performed by inserting a catheter into either (1) the nostril or (2) the mouth to reach into the posterior pharynx or to the proximity of the open trachea, withdrawing the catheter while applying a gentle suction with an electric suction device [4–6]. An induced sputum sample can be performed in many ways, usually by pre-treatment of inhaled Salbutamol and hypertonic saline solution via a nebulizer for minimum of 10 min. followed by oropharyngeal suction by catheter inserted into the mouth to reach into the posterior pharynx or to the proximity of the open trachea, withdrawing the catheter while applying a gentle suction with an electric suction device [7–9]. The validity of these methods has been investigated in various settings with different results [2–9].

In 1995, Avital et al. in a small study sample concluded that NTA may be helpful in the diagnosis and treatment of recurrent or chronic pulmonary infection [6]. However, the following studies (Asseri et al. [2], Hare et al. [3], Lu et al. [5]) concluded that a negative swab and/or negative NPA/NTA result reduces the likelihood of lower airway infection. A positive sample, however, does not accurately predict the presence of lower airway pathogens [2, 3, 5]. All previous studies have attempted to validate the above-mentioned methods by comparing with BAL in intubated patients. It remains uncertain if results on aspiration in intubated children are transferable to non-intubated children. Endotracheal intubation enables direct sampling from the trachea; however, the tube or a laryngeal mask is a foreign body and may be a route of contamination from the upper to the lower airways when installed. All of the above-mentioned methods were performed as blind procedures, which complicates the examination and increases the possibility of inaccuracy. Kim et al. have developed an algorithm to predict the nares vocal cord distance (NV distance) by external measurements and calculations to make the procedures more accurate [10]. This algorithm has not been included in any previous study comparing upper airway sampling to the gold standard BAL as attempted in this study.

Furthermore, the microbiota varies between the upper and lower airways [11–15], which could lead to false microbiological results in case of contamination and faulty placement. Studies concerning non-invasive lower airways sampling methods are of interest to clinical paediatricians as this topic is an ongoing and recurring challenge in children.

To the best of our knowledge, our study is the first study comparing cultures obtained from NTA taken by specific NV distance and BAL in non-intubated patients to validate the usage of NTA.

We hypothesised that NTA performed by health professionals using the nares vocal cord distance to be placed at the entrance of the trachea, will result in same finding microbiological results in the lower airways as BAL.

## Methods

This prospective cohort study was conducted between June 2016 and August 2018 at the Department of Paediatrics and Adolescent Medicine, Center of Pediatric Pulmonology and Allergology, Aarhus University Hospital, Denmark. All patients referred to bronchoscopy for diagnostic or interventional purposes were included in the study. In total, 173 consecutive procedures were performed during the study period with no exclusions. The 173 paired examinations were obtained in 164 patients and a few children underwent more than one paired examination during the study period. All samples were obtained using the same procedure.

All children were included regardless race, ethnicity, gender and age; thus representative to the Danish population. The included children are primarily generalizable to other children in need for undergoing investigations for pulmonary diseases.

All parents or guardians provided verbal consent prior to the examinations.

The examinations were performed in general anaesthesia on spontaneous breathing. NTA was performed as the first procedure after induction of general anaesthesia before bronchoscopy.

The NTA sample was collected by inserting a catheter through the nostril to reach to the proximity of the open trachea and performed blindly using the NV distance algorithm [10]; subsequently, a gentle suction with an electric suction device was performed mixing aspirate with a maximum of 1 mL sterile isotonic sodium chloride in a transport tube immediately forwarded to laboratory analysis. The bronchoscopy was performed through the naris according to international guidelines, performing BAL in one lobe using lavage-1 for bacterial culture [16]. The BAL sample was collected as the first thing in a specific lobe by clinical indication; otherwise in the right middle lobe. A total of approximately 2 ml/kg and maximum 40 mL sterile isotonic sodium chloride was installed and aspirated again from the lobe of investigation.

All examinations were performed by the same three experienced senior pediatric pulmonologists. Furthermore, all data were continuously validated by the head pulmonologist for within-sample repeatability during the prospective study.

The samples from both the NTA and BAL procedures were cultured according to standard procedures at Department of Clinical Microbiology, Aarhus University Hospital. The samples were handled as all other samples in the hospital. The laboratory technicians were not informed about the study. Thus, the paired samples are most likely counted by the same lab technician, but this cannot be confirmed.

The results were described as the presence of different bacteria. Our study compared the results of the bacteria from cultures obtained by the paired NTA and BAL examinations.

### Data collection

After conclusion of this prospective study, all data were extracted from the electronic patient records.

The following data were registered for every paired sample.

- Relevant diagnostic codes (up to eight different codes).
- Microbiological culture and antibiotic resistance in the paired samples.
- Whether or not antibiotic treatment had been initiated.

For the purpose and clinical relevance of this study, we decided that only pathogen microorganisms should be included in the statistical analysis. Apathogenic and commensal microorganisms (*Coagulase negative Staphylococcus*, *Non Hemolytical Streptococcus*, *Corynebacterium species* and *Neisseria species*) were excluded from analysis because the hospital guideline for analysis of airway secretion does not clarify these in NTA sampling and these bacteria are not deemed necessary to initiate antibiotic treatment.

The NTA count was semi-quantitative and followed guidelines at Department of Clinical Microbiology. Thus, laboratory technicians or microbiologists performed an individual assessments of the number of bacteria. The NTA was described as few (1–4), scattered (5–49), some (50–499) or many (> 500) colonies.

The BAL count was quantitative and expressed as colony forming unit (CFU)/mL.

Our study focused on bacteria causing possible lower airway infection. Previous studies found a threshold ≥ 10^4^ CFU/mL to define lower airway infection in children with endobronchial disorders [17] and ≥ 10^5^ CFU/mL for children with cystic fibrosis (CF) [13].

In our study, a wide range of diagnoses were included, meaning that great attention was given to the inclusion of bacteria. The primary and secondary pathogens were registered regardless of number of CFU/mL except from *Hemolytical Streptococcus Group A*, *Enterobacteriaceae gram negative rods* and *Escherichia Coli.* These bacteria were only registered if the number was > 10^4 CFU/mL in BAL or > 49 colonies in the NTA, because the bacteria pathogenesis may cause diseases when the count of bacteria count exceeds these values.

Registration of the paired NTA and BAL led to two different classifications from which the statistics were calculated:

- Concordance bacteria (previously described in reference [15]):
  - 1) if both samples found an identical match of pathogenic bacteria culture/cultures (if more than one type of bacteria was found – all bacteria culture had to be identical)
  - 2) if no bacteria were found in both samples
- Coherence growth meaning:
  - 1) both samples had pathogenic bacteria but the type/types and/or number varied and were not completely identical
  - 2) no bacteria were found in both samples

The samples were subdivided into four different groups by diagnosis: CF, rare lung diseases, airway malformation and other lung-related diseases (Appendix 1). Samples from patients with several diagnoses were placed under the group of highest priority: (1) CF, (2) Rare lung diseases, (3) Airway malformation and (4) Other lung-related diseases.

### Statistics

Similarity, specificity, sensitivity, positive predictive value and negative predictive value for NTA were calculated using standard formulas (2 × 2 table) with BAL as the gold standard.

When calculating the concordance of bacteria, we excluded the samples with a positive different growth composition. This group had a positive BAL and NTA, but concordance was not complete, thus being placed in the denominator when calculating sensitivity and positive predictive value.

Calculations were performed in diagnosis groups (all samples, CF, rare lung diseases, airway malformation and other lung-related diseases), antibiotic treatment at the time of examination and for the samples when common upper airway tract bacteria were omitted.

The statistics concerning diagnosis compared to other diagnoses were based upon data presented in Table 3 and subsequently calculating p-values were calculated to find out if differences between the diagnoses were statistically significant.

To calculate the 95% confidence intervals we used the Wilson/Brown Method. The p-values were calculated using the Chi-squared test.

Microsoft Excel for Mac 2011, version 14.5.2 and Prism 8 were used for statistical analysis.

### Power analysis

Considerations regarding whether the study populations would have a sufficient size to answer the stated study aims, a power analysis was performed by sample Size Calculator (https://clincalc.com/stats/samplesize.aspx). There are only few published studies investigating clinical outcomes on NTA compared to BAL. In these consistency between NTA and BAL was found in 36% regarding the infectious agent in intubated children [2]. We hypothesised that NTA performed by health professionals using the nares vocal cord distance placed at the entrance of the trachea, would show the same results BAL. Thus, yielding a clinically significant and relevant similarity between NTA and BAL of at least 80%. Power analysis with α = 0.05 and power = 95% showed that the study required a sample size of 46 in total. The study included a population size of 174 paired samples and was deemed sufficiently powered.

## Results

A total of 173 paired samples were obtained from 164 patients (158 children between 21 days to 18 years of age and six young adults still treated at the department).

The most common diagnosis was DJ984 “other lung disease”, which is a combined diagnosis code used for unknown lung disease. The second most common was DQ339 “congenital lung malformation”. In total, 75 different diagnoses were registered with some overlap of several diagnoses per child. In 33 cases, the samples were obtained from patients with CF and in 75 cases sampling was obtained from patients with different types of airway malformations. Demographics of the samples investigated are listed in Table 1.

**Table 1:** Demographic data of the samples

Parameter	Number	
Number of samples	173	
Male/females ratio	94/79	
Age		
6 paired samples were performed in patients > 18 years and 6 young adults still treated at the paediatric department	21 days–27 years	

Diagnosis*		Number (%)
DJ984	Other rare lung disease	109 (63%)
DQ339	Congenital lung malformation	70 (42%)
DQ338	Other congenital lung malformation	62 (36%)
DJ459	Asthma	57 (33%)
DQ349	Congenital malformation in the respiratory system	34 (20%)
DE849	Cystic fibrosis	33 (19%)
DJ981	Atelectasis in the lung	33 (19%)
DQ249	Congenital heart malformation	20 (12%)
DQ320	Tracheomalacia congenita	14 (8%)
DQ318H	Laryngeal condromalacia	9 (5%)
D849	Immunodeficiency	8 (5%)

*The 11 most common registered diagnosis

The following bacteria were found in the samples and evaluated as pathogenic by a senior consultant from Department of Clinical Microbiology as pathogenic. The number in the brackets indicates how many samples found with the pathogen: *Staphylococcus aureus (53), Haemophilus influenza “H. influenza” (82), Streptococcus pneumonia “S. pneumonia” (36), Haemolytic streptococcus group A (4), Moraxella catarrhalis “Moraxella” (56), Achromobacter species (2), Pseudomonas aeruginosa (27), Escherichia Coli (5), Stenotrophomonas maltophilia (9), Bordetella (2), Haemophilus parahemolyticus (2), Rothia mucilaginosa (1), Serratia marcescens (1), Nocardia species (2), Meningococcus (1), Citrobactor freundii (1), and Burkholderia cepacia group (2)*, *Enterobacteriaceae gram negative rods (7)* and *Klebsiella pneumonie (1).*

The complete bacteria concordance between NTA and BAL is listed in Table 2 and was found in 84 of the 173 samples (48.6%) [41–56 (95% CI)] of which 49 were true negative (no bacteria culture in both samples) and 35 were true positive; thus, NTA resulted in the exact same bacteria culture/cultures as BAL.

**Table 2:** Complete similarity between NTA and BAL (all samples)

NTA similar to BAL	NTA was not similar to BAL
84 (35 true positive) (49 true negative)	89 (26 with positive BAL and negative NTA) (25 with positive NTA and negative BAL) (38 with different positive culture)

*NTA* nasal tracheal aspiration, *BAL* bronchoalveolar lavage

For complete concordance, the sensitivity of NTA was 35% [27–45], specificity was 66% [55–76], positive predictive value was 36% [27–46] and negative predictive value was 64% [54–64].

In 89 cases, the samples were not completely concordant. Of these, 38 samples had positive culture in both NTA and BAL, but the types of bacteria varied. We also calculated sensitivity, specificity, positive predictive value and negative predictive value for coherence of growth between NTA and BAL not considering type of bacteria; here we found a similarity of 71% between samples [63–79] (Table 3).

**Table 3:** Diagnosis, antibiotic treatment and bacteria—Sim, SN, SP, PPV, NPV compared to all samples

	NTA					p-value
		Positive	Negative	Results	95% CI	
Statistics by diagnosis						
***All samples (N*** **= *173)***						
*Coherence growth*^a^						
BAL	Positive	73	26	Sim: 122 (71%)	63–79	
	Negative	25	49	SN: 74%	64–81	
				SP: 66%	55–76	
				PPV: 75%	65–82	
				NPV: 65%	54–75	
*Concordance bacteria*^b^						
BAL	Positive	35	26	Sim: 84 (49%)	41–56	
	Negative	25	49	SN: 35%	27–45	
	38 samples had different growth composition			SP: 66%	55–76	
				PPV: 36%	27–46	
				NPV: 64%	54–64	
***Cystic Fibrosis (33)***						
*Coherence growth*						
BAL	Positive	24	2	Sim: 29 (88%)	73–95	(**0.036**)^**c**^
	Negative	2	5	SN: 92%	76–99	(**0.025**)
				SP: 71%	36–95	(0.58)
				PPV: 92%	76–99	(**0.025**)
				NPV: 71%	36–95	(0.51)
*Concordance bacteria*						
BAL	Positive	13	2	Sim: 18 (55%)	38–70	(0.50)
	Negative	2	5	SN: 50%	32–68	(0.10)
	11 samples had different growth composition			SP: 71%	36–95	(0.58)
				PPV: 50%	32–68	(0.13)
				NPV: 71%	36–95	(0.51)
***Rare lung diseases (36)***						
*Coherence growth*						
BAL	Positive	12	6	Sim: 26 (72%)	56–84	(0.84)
	Negative	4	14	SN: 67%	44–83	(0.39)
				SP: 78%	55–91	(0.16)
				PPV: 75%	51–90	(0.90)
				NPV: 70%	48–86	(0.56)
*Concordance bacteria*						
BAL	Positive	4	6	Sim: 18 (50%)	35–66	(0.88)
	Negative	4	14	SN: 22%	9–45	(0.13)
	8 samples had different growth composition			SP: 78%	55–91	(0.16)
				PPV: 25%	10–50	(0.21)
				NPV: 70%	48–86	(0.56)
***Airway malformation (75)***						
*Coherence growth*	Positive	29	14	Sim: 47 (63%)	51–73	(0.26)
	Negative	14	18	SN: 67%	53–80	(0.36)
				SP: 56%	40–72	(0.13)
				PPV: 67%	53–80	(0.26)
				NPV: 56%	40–72	(0.18)
*Concordance bacteria*	Positive	11	14	Sim: 29 (39%)	29–50	(0.16)
	Negative	14	18	SN: 26%	15–40	(0.16)
	18 samples had different growth composition			SP: 56%	40–72	(0.13)
				PPV: 26%	15–40	(0.12)
				NPV: 56%	40–72	(0.18)
***Other lung-related diseases (29)***						
*Coherence growth*						
BAL	Positive	8	4	Sim: 20 ( 69%)	51–83	(0.89)
	Negative	5	12	SN: 67%	39–86	(0.43)
				SP: 71%	47–87	(0.6)
				PPV: 62%	36–82	(0.18)
				NPV: 75%	51–90	(0.29)
*Concordance bacteria*						
BAL	Positive	7	4	Sim: 19 (66%)	47–80	(0.083)
	Negative	5	12	SN: 58%	32–81	(**0.018**)
	1 samples had different growth composition			SP: 71%	47–87	(0.6)
				PPV: 54%	29–77	(0.06)
				NPV: 75%	51–90	(0.29)
Statistics concerning antibiotic treatment						
***Sample on antibiotics***^e^ ***(58)***						
*Coherence growth*						
BAL	Positive	23	5	Sim: 42 ( 72%)	60–82	(0.81)
	Negative	11	19	SN: 82%	64–92	(0.21)
				SP: 63%	46–78	(0.68)
				PPV: 68%	51–81	(0.38)
				NPV: 79%	60–91	(**0.047**)
*Concordance bacteria*						
BAL	Positive	13	5	Sim: 32 (55%)	43–67	(0.40)
	Negative	11	19	SN: 46%	30–64	(0.13)
	10 samples had different growth composition			SP: 63%	46–78	(0.68)
				PPV: 38%	24–55	(0.78)
				NPV: 79%	54–91	(**0.047**)
***Sample with no antibiotics (115)***						
*Coherence growth*						
BAL	Positive	50	21	Sim: 80 (70%)	61–77	(0.96) [0.79]^d^
	Negative	14	30	SN: 70%	59–80	(0.48) [0.09]
				SP: 68%	53–80	(0.72) [0.51]
				PPV: 78%	67–87	(0.44) [0.15]
				NPV: 59%	45–71	(0.30) [**0.009**]
*Concordance bacteria*						
BAL	Positive	22	21Uden	Sim: 52 (45%)	36–54	(0.55) [0.21]
	Negative	14	30	SN: 31%	21–43	(0.48) [**0.05**]
	28 samples had different growth composition			SP: 68%	32–53	(0.72) [0.51]
				PPV: 34%	24–47	(0.72) [0.60]
				NPV: 59%	25–71	(0.30) [**0.009**]
Statistics for all samples when common upper airway tract bacteria omitted						
***All samples—without H. influenza***^f^						
*Concordance bacteria*						
BAL	Positive	35	26	Sim: 98 (57%)	49–64	(0.12)
	Negative	11	63	SN: 35%	27–45	(1.00)
	38 samples had different growth composition			SP: 85%	75–92	(**< 0.001**)
				PPV: 42%	32–52	(0.25)
				NPV: 71%	61–79	(0.23)
***All samples—without Moraxella***^f^						
*Concordance bacteria*						
BAL	Positive	40	26	Sim: 93 (54%)	46–61	(0.32)
	Negative	21	53	SN: 40%	31–50	(0.34)
	33 samples had different growth composition			SP: 72%	61–81	(0.23)
				PPV: 43%	33–53	(0.18)
				NPV: 67%	56–76	(0.69)
***All samples—without S. pneumonia***^f^						
*Concordance bacteria*						
BAL	Positive	37	26	Sim: 88 (51%)	44–58	(0.66)
	Negative	23	51	SN: 37%	29–47	(0.70)
	36 samples had different growth composition			SP: 69%	58–78	(0.55)
				PPV: 39%	29–49	(0.56)
				NPV: 66%	55–76	(0.84)
***All samples—without H. influenza or Moraxella***^g^						
*Concordance bacteria*						
BAL	Positive	40	25	Sim: 107 (62%)	54–69	(**0.012**)
	Negative	7	68	SN: 41%	32–51	(0.25)
	33 samples had different growth composition			SP: 91%	82–95	(**< 0.001**)
				PPV: 50%	39–61	(**0.009**)
				NPV: 73%	63–81	(0.11)

Bold: *p*-values ≤ 0.05 are statistically significant

*NTA* nasal tracheal aspiration, *BAL* bronchoalveolar lavage, *CI* confidence interval, *Sim* similarity, *SN* sensitivity, *SP* specificity, *PPV* positive predictive value, *NPV* negative predictive value, *H. influenza*: *Haemophilus influenza*, *Moraxella: Moraxella catarrhalis*, *S. pneumonia: Streptococcus pneumonia*

^a^Coherence growth: Agreement is only based on positive growth or not in NTA and BAL

^b^Concordance bacteria: Complete concordance concerning pathogen bacteria between NTA and BAL

^c^The (p-values) are calculated in relation to”All samples”

^d^The [p-value] are calculated in relation to”with antibiotics”

^e^The samples are placed in this group if the patient received antibiotics less than or three days before the examination or during the examinations—except if the treatment started the same day

^f^If the bacteria was the only difference between the culture in NTA and BAL it was removed for the statistical analysis

^g^If one of *H. Influenza* or *Moraxella* was the only difference between the culture in NTA and BAL it was removed for the statistical analysis

The results for sensitivity, specificity, positive predictive value, negative predictive value and related p-values in the subdivided groups are listed in Table 3. The p-values were calculated in relation to “all samples” to find if the results from the subdivided groups were statistically significantly different from all samples. NTA showed the best results in patients with CF with a statistically significantly better similarity (p = 0.036), sensitivity (p = 0.025) and positive predictive value (p = 0.025) compared to “all samples”. The NTA from patients with airway malformations showed the least valid results, though not statistically significantly different from “all samples”.

Statistics comparing the result of NTA in the different diagnosis groups are listed in Table 4. NTA performed in patients with CF showed statistically significantly better results regarding sensitivity and positive predictive value than the other three diagnosis groups.

**Table 4:** Statistics by diagnosis compared to other diagnoses

	Similarity	Sensitivity	Specificity	PPV	NPV
*CF >< Rare lung disease*					
Coherence growth^a^	**0.099**	**0.011**	0.504	0.060	0.928
Concordance bacteria^b^	0.678	**0.015**		**0.032**	
*CF >< Airway malformation*					
Coherence growth	**0.009**	**0.006**	0.141	**0.006**	0.142
Concordance bacteria	0.123	**0.015**		**0.015**	
*CF >< other lung-related disease*					
Coherence growth	0.066	**0.014**	1	**0.004**	0.724
Concordance bacteria	0.377	0.528		0.753	
*Rare lung disease >< Airway malformation*					
Coherence growth	0.349	1	**0.025**	0.391	0.158
Concordance bacteria	0.273	0.647		0.910	
*Rare lung disease >< other lung-related disease*					
Coherence growth	0.792	1	0.518	0.260	0.655
Concordance bacteria	0.195	**0.003**		**0.017**	
*Airway malformation >< other lung-related disease*					
Coherence growth	0.57	1	0.161	0.630	0.074
Concordance bacteria	**0.01**	**0.002**		**0.007**	

Bold: *p*-values ≤ 0.05 are statistically significant

*PPV* positive predictive value, *NPV* negative predictive value

^a^Coherence growth: Agreement is only based on positive growth or not in NTA and BAL

^b^Concordance bacteria: Complete concordance concerning pathogen bacteria between NTA and BAL

A total of 89 of the samples were not completely concordant concerning the exact same bacteria culture/cultures. In 44 samples, BAL found a higher number of different bacteria than NTA. In 63 of the 89 samples, the difference was caused by one bacteria. In 29 of these 63 cases, the NTA found one more bacteria than BAL. Table 3 lists the similarity, sensitivity, specificity, positive predictive value and negative predictive value if common upper airway tract bacteria (*H. influenza*, *Moraxella*, *S. pneumonia*) found in the NTA were left out of the statistical analysis between BAL and NTA. After removing *H. influenza*, the result showed a statistically significant improvement of specificity of 85% [75–92] against 66% [55–76] for all samples (p < 0.001). Furthermore, the similarity of 62% [54–69] (p = 0.012), specificity of 91% [82–95] (p < 0.001) and the positive predictive value of 50% [39–61] (p = 0.009) were all statistically significantly improved when leaving out both *H. influenza and Moraxella* from the analysis.

Some of the children were treated with antibiotics at the time of examination; thus, 58 paired samples were performed on patients already receiving antibiotics. Table 3 describes the statistics according to antibiotic treatment before the examinations. Sensitivity of the concordance of the bacteria without antibiotics was 31% [21–43] and for the patients treated with antibiotics it was 46% [30–64]; however, the p-value was 0.05 resulting in a statistically significant difference. The negative predictive value increased statistically significantly for patients receiving antibiotics with 79% [54–91] (p = 0.009) against 59% [25–71] for those not treated with antibiotics.

## Discussion

### Main findings

A total similarity between the bacteria concordance of NTA and BAL was only found in 49% [41–56] of the samples, which rejected our hypothesis. Thus, this study indicated that NTA and BAL were not interchangeable diagnostic tools for microbiota in the lower airway tract.

Corresponding to the exact bacteria concordance, sensitivity was 35% [27–45] and the positive predictive value was 36% [27–46]. Specificity and negative predictive value were higher, 66% [55–76] and 64% [54–64], respectively.

Concerning coherence of growth of pathogen bacteria, the sensitivity was 74% [64–81] and the positive predictive value was 75% [65–82]. Specificity and negative predictive value were 66% [55–76] and 65% [54–75], respectively.

Our study suggests that the clinical usage of microbiota from NTA was most reliable if the culture was negative. However, if NTA was used to indicate if the lower airway tract was colonized with pathogenic bacteria, the agreement of culture growth was 71% [63–79] and sensitivity was 74% [64–81] for the lower airway tract.

To the best of our knowledge, our study is the first to compare cultures obtained from NTA taken by specific NV distance and BAL in non-intubated patients. Therefore, we find this study relevant for clinical paediatricians, when determining whether to use the BAL to obtain valid positive findings or to use NTA with its usability to detect negative findings.

### Strengths and limitations

This study compared cultures obtained from NTA and BAL in non-intubated patients from a large population group of 173 samples. The examinations were performed in a selective group of symptomatic children with different lung diseases considered representative for children with a possible or diagnosed lung disease. Thus, sampling results cannot necessarily be transferred to asymptomatic children.

The method used for the NTA was standardized and performed under optimal conditions. (1) The NTA was obtained using the NV distance algorithm, which minimizes the possibility of misplacement in the airway tract, (2) All children were in general anaesthesia eliminating undesirable head movements, and (3) All examinations were performed by the same three experienced senior paediatric pulmonologists. Within-sample repeatability was ensured by always having two of the three pulmonologists in the facility at all times agreeing how to perform the NTA and BAL thus ensuring uniform procedures.

Furthermore, all data were continuously validated by the head pulmonologist for within-sample repeatability and no intra-individual variability between the three pulmonologists was found, which could have impacted on results.

As no tube or laryngeal mask was used during the anaesthesia, we believe the algorithm for obtaining NTA is as close to clinical reality as possible and thus the most accurate clinical approach.

BAL was obtained according to international guidelines [16]. A previous study found that more bacteria were found in the lungs if more than one lobe was investigated [18] and if lavage-2 was also cultured for bacteria [19].

This means that BAL as performed in our study is the gold standard, though not necessarily representing the complete bacterial composition in the lungs.

### Detailed findings

As the NV distance is calculated from the algorithm and used during the procedure the possibility of wrong insertion and placement of the NTA tube was prevented. The false results of NTA might be explained by contamination from the upper airway tract during the examination, wrong placement of the aspiration or that the area just above rima glottides is not comparable to the lower airways.

Zhang et al. performed nasopharyngeal swabs and nasotracheal aspiration in a group of children below three years and found af false positive rate of 27% for S. pneumonie, 22.1% for Moraxella and 8.4% for S. pneumonia [20]. Thus, Zhang et al. confirms that when NTA is performed, the risk of contamination from the upper airways is present. Application of methods such as NV distance calculation should be used in order to minimize this risk. Thus, the importance of NTA finding the correct microbiota is questioned. BAL is the gold standard and the bronchoscopy is validated to investigate the lung microbiota with little risk of contamination [21].

Hare et el investigated cultures from oropharyngeal and nasopharyngeal swabs from 309 children with chronic cough. Their study showed that neither nasopharyngeal nor oropharyngeal swabs, alone or in combination, reliably predicted lower airway infection compared to BAL in children, which is in line with our study results [8].

McCauley et al. investigated 84 intubated adults with a clinically and radiographically verified pneumonia [22]. A tracheal aspiration right after intubation found a pulmonary pathogen in 47 of 84 patients concluding that tracheal aspirate culture offers important additive diagnostic value compared to other routine tests. However, this study did not compare findings to real BAL as the gold standard [22]. Furthermore, the study [22] was conducted in intubated patients and thus not applicable as a “routine test”, whereas our study compared NTA standard to BAL results, which is more generalizable to children in broader clinical situations with non-intubated children.

To evaluate potential contamination we made statistical calculations removing common upper tract airway tract bacteria such as *H. influenza*, *Moraxella* and *S. pneumonia* if one of these bacteria was the only difference between the microbiota in NTA and BAL cultures. *H. influenza*, *Moraxella* and *S. pneumonia* are all potentially important pathogens in the lower airways in children, but are also known as common upper airway tract bacteria. Only if these bacteria were found in BAL in high amounts (very high CFU), they were considered indicative of antibiotic treatment.

The removal of *H. influenza* made a statistically significant change of the specificity to 85% [75–92]. Both the similarity, specificity and the positive predictive values were statistically significantly increased when *H. Influenza* or *Moraxella* were removed. We do not suggest ignoring the findings of *H. influenza* or *Moraxella*, but in a clinical perspective we find a specificity of 91% [82–95] interesting, if a potential contamination from H. influenza or Moraxella is removed, which was the only difference between the cultures in NTA and BAL in our study.

When subdividing into diagnosis groups (Appendix 1), especially the diagnosis of airway malformations had a statistically significantly poor result on different parameters compared to CF, other lung-related diseases and rare lung diseases (Table 4). A possible medical explanation for this could be a higher risk of accumulation of bacteria in the lower airway tract with the lack of ability to mobilise the bacteria upwards because of anatomical structural changes. This study suggests that usage of NTA in children with airway malformations is not recommendable or that results should be interpreted with clinical caution.

Compared to the overall NTA, the results from the CF group appeared to be more in agreement with BAL.

It is of potential clinical importance that NTA cultures from CF provided better results with the paired BAL sample as patients with CF often need an assessment of bacteria in the airways followed by adequate antibiotic treatment. The increased and statistical significant sensitivity of 92% [76–99] concerning coherence growth might correspond to the tenacious mucus in patients with CF, which leads to widely spread colonization in the entire airway system.

Antibiotic treatment at the time of the examination seemed to have a positive impact on the sensitivity of NTA. A possible explanation could be that antibiotics remove the normal flora and thereby minimize contamination and exposure of the upper airway tract with pathogen bacteria. These changes are close to being statistically significant with p-values of 0.09 and 0.05.

### Future clinical and research implications

This study recommends BAL for highly accurate sampling of specific bacteria in the lower airway tract. However, it is not clinically feasible to only use BAL as a reliable diagnostic tool to detect agents causing lower tract airway infections, due to the invasive nature of the procedure requiring children to undergo general anaesthesia.

Further research is needed to confirm whether the inexpensive, feasible and accessible NTA is a useful diagnostic tool in non-intubated children to determine pathogens and thus select appropriate antibiotic treatment.

Man et al. suggest that nasopharyngeal microbiota can serve as a valid proxy for lower respiratory tract microbiota in lower respiratory tract infections as they found the concordance high between nasopharyngeal and endotracheal aspirate samples on intubated children [23]. Our and previous other studies [8–10] questions this conclusion, as we found the NTA and BAL not to be interchangeable diagnostic tools, no matter whether the method to indicate lower respiratory tract infection was standard bacterial growth or quantitative PCR and 16S rRNA-based sequencing.

The most encouraging non-invasive procedure may be induced sputum [9]. The procedure of induced sputum is lengthy and lasts approximately about 30 min [8]. Despite the non-invasive nature of the method, it is considered harsh and not possible to be used in smaller children (below age 10 years) as they cannot expectorate. Furthermore, the issue of contamination through the upper airways may also be considered to influence the validity of this procedure. Therefore, further research concerning feasibility of the induced sputum procedure in children with lung diseases is needed.

We conclude that NTA taken by specific NV distance and BAL are not interchangeable diagnostic tools to detect microbiota in the lower airway tracts. Our study indicates that NTA is not clinically useful to assess specific bacteria in the lower airway tract. Statistically significantly increased sensitivity and positive predictive value were found in patients with CF, which may be clinically relevant in the monitoring of children with CF.

Furthermore, the clinical usage of NTA in general remains important, as a negative NTA results reduces the likelihood of lower airway infection. The BAL sampling method is the "gold standard" and preferred to NTA when accurate bacteria-specific diagnostics of lower airway tract infections is needed.

## Data Availability

The dataset used and analysed during the current study are available from the corresponding author on request.

## Appendix 1

**Table:**

Diagnosis code	Diagnosis	Diagnosis code	Diagnosis
**1. Cystic fibrosis**		**4. Other lung-related diseases**	
DE849	Cystic fibrosis	DJ450–DJ459	Asthma
**2. Rare lung diseases**		DJ981	Atelectasis
DJ849	Interstitial lung disease	DQ391	Oesophageal atresia with trachea-esophageal fistula
DJ988A	Isolated ciliary dyskinesia	DP220	Idiopathic infant respiratory distress syndrome
DJ982	Interstitial lung emphysema	DP271	Bronchopulmonal dysplasia arisen in the perinatal periode
DJ961	Chronic respiratory insufficiency	DP271A	Moderate bronchopulmonal dysplasia arisen in the perinatal periode
DD849	Immunodeficiency	DJ809	Damage of the alveoli with respiratory failure (ARDS)
DJ479	Bronchiectasis	DQ790	Congenital diaphragmatic hernia
DJ841	Other interstitial lung illness with fibrosis	DJ448B	Chronic asthmatic bronchitis
DJ429	Chronic bronchitis	DJ980D	Tracheal stenosis
DJ84	Other interstitial lung diseases	DJ840	Alveoli or parieto-alveolar disease
DJ841C	Idiopathic lung fibrosis	DQ336	Hyperplasia or dysplasia of the lung
DQ334	Congenital bronchiectasis	DD143A	Benign tumour in bronchia
DJ848A	Allergic bronchopulmonal aspergillosis	DP288	Other congenital airway disease
DD475	Hypereosinophilic syndrome	DQ391A	Oesofageal atresia with congenital broncho? esophageal fistula
		DJ984	Other lung disease
**3. Airway malformation**			
DQ338	Other congenital lung malformation	DQ322	Bronchomalacia congenita
DQ339	Congenital lung malformation	DQ349	Congenital malformation in the respiratory system
DJ399	Illness in the upper respiratory system	DQ321	Other congenital malformation in trachea
DQ322	Tracheal bronchomalaci	DJ39	Other diseases in the upper respiratory system
DQ321B	Tracheal condromalaci	DQ324	Other congenital bronchial malformation
DQ320	Tracheomalacia congenita	DJ386	Laryngo stenosis
DQ318H	Laryngeal condromalacia	DJ383V	Vocal cord dysfunction

## Abbreviations

AB: Antibiotics
BAL: Bronchoalveolar lavage
CF: Cystic fibrosis
CFU: Colony forming units
*H. influenza*: *Haemophilus influenza*
*Moraxella*: *Moraxella catarrhalis*
NPV: Negative predictive value
NTA: Nasal tracheal aspiration
NV distance: Nares vocal cord distance
PPV: Positive predictive value
Sim: Similarity
SN: Sensitivity
SP: Specificity
*S. pneumonia*: *Streptococcus pneumonia*
